# Diminished Social and Leisure Engagement in Community Dwelling-Older Adults with Apathy

**DOI:** 10.3390/ijerph22071138

**Published:** 2025-07-18

**Authors:** Katherine J. Valles, Emmeline Ayers, Joe Verghese, Mirnova E. Ceïde

**Affiliations:** 1 Department of Psychiatry, Eastern Connecticut Health Network, Manchester, CT 06040, USA; katherinevalles@gmail.com; 2 Division of Cognitive and Motor Aging, Albert Einstein College of Medicine, Bronx, NY 10461, USA; emmeline.ayers@stonybrookmedicine.edu (E.A.); joe.verghese@stonybrookmedicine.edu (J.V.); 3 Department of Neurology, Renaissance School of Medicine at Stony Brook University, Stony Brook, NY 11794, USA; 4 Department of Psychiatry and Behavioral Sciences and Medicine, Montefiore Medical Center, Bronx, NY 10461, USA

**Keywords:** social engagement, loneliness, cognition, leisure activity, physical activity

## Abstract

Background: Apathy predicts functional and cognitive decline in community-dwelling older adults. However, the behavioral correlates of apathy, which could promote cognitive decline have not been described. Our objective was to investigate the associations of apathy with leisure and social engagement. Methods: *N* = 538 older adults enrolled in the Central Control of Mobility in Aging study. We used the GDS3A, a 3-item subscale of the Geriatric Depression Scale, to define apathy and the frequency of participation in cognitive, physical, and social leisure activities. Linear regression models were conducted to assess the association between apathy and its behavioral correlates: social engagement and leisure activity participation. Covariates included age, gender, education level, multimorbidity, and dysphoria. Results: Apathy was present in 29.7% of participants and was significantly associated with less frequent participation in physical activity days per week (−1.688. *p* = 0.003) but not cognitive (−1.094, *p* = 0.252) or social (−0.654, *p* = 0.103) leisure activities. Apathy was also associated with a decreased social behavior composite score (−0.055, *p* < 0.001), Social Network Index (−0.478, *p* = 0.003), and Medical Outcomes Study Social Support scores (−0.26, *p* = 0.001). Conclusions: Our findings suggest that apathy presents with reduced participation in physical leisure activities and reduced social engagement, which may provide a way for clinicians and caregivers to identify apathy in the future.

## 1. Introduction

Apathy is a neuropsychiatric syndrome of diminished motivation that is characterized by deficits in the cognitive, emotional, and behavioral domains of goal-directed behavior [1,2]. Largely studied in the context of neurologic disease, apathy is highly prevalent in dementia, stroke, and Parkinson’s disease (PD) and is associated with poorer cognitive and functional outcomes [3,4,5]. A growing body in the literature has begun to investigate apathy in community-dwelling older adults. Apathy becomes more prevalent with advancing age, with reported rates in community-dwelling older adults without dementia ranging from 10% to 30% [6,7,8]. Apathy in this population is associated with worse cognitive performance, functional impairment, depressive symptoms [9], disability, and diminished overall quality of life [10,11]. Longitudinal studies of community-dwelling older adults have further demonstrated that apathy predicts the incidence of physical disability [7], functional and cognitive decline [6], pre-dementia syndromes [12], and dementia [13,14].

Apathy is increasingly being recognized as a distinct and persistent syndrome that significantly affects quality of life, morbidity, and mortality in older adults, independent of depression, and is considered a high-value neuropsychiatric risk state according to the National Institute on Aging–Alzheimer’s Association workgroups on the diagnostic guidelines for Alzheimer’s disease, given its overwhelming association with mild cognitive impairment, dementia, and Alzheimer’s disease biomarkers [15]. Recent studies indicate that apathy mediates the association between depression and cognitive decline in PD [16] and the functional decline in persons with stroke and traumatic brain injury [17]. Funes and colleagues report that apathy mediates the relationship between depression and cognitive impairment [18]. Similarly, van Dalen and colleagues found that the apathy-related items of the Geriatric Depression Scale (GDS), but not the remaining 12 depression-related items [19], were significantly associated with incident dementia, findings that were later reproduced in our CCMA cohort [12].

Investigations into the mechanistic pathway between apathy and dementia are limited and have largely focused on physiological factors. Apathy is associated with an increased risk of cardiovascular disease and biomarkers suggesting an underlying inflammatory state [20,21], and the presence of deep white matter lesions in the brain mediate the relationship between apathy and inflammation [22]. Decreased motor activity has been observed with apathy in people with schizophrenia [23], as well as older adults with cognitive impairment [24] and Alzheimer’s disease [25]. Though studies in non-demented community-dwelling older adults are limited, a similar relationship has been shown where apathy is associated with decreased physical activity but was not independent of depression in one study [26], and that apathy may mediate the relationship between white matter brain lesions and leisure time physical inactivity [27].

There is accumulating evidence that apathy may be an early behavioral manifestation of the underlying brain dysfunction in dementia that precedes cognitive and motor impairment. However, the behavioral pathway between apathy and cognitive decline has been minimally explored. The objective of our study was to explore the behavioral correlates of apathy in community-dwelling older adults. By focusing on leisure activity and social engagement, we investigated how apathy relates to volitional engagement in rewarding behavior. We examined the cross-sectional associations of apathy with participation in physical, cognitive, and social leisure activity. We also explored associations of apathy with social behavior, including engagement with social network and perceived availability of social support.

## 2. Materials and Methods

### 2.1. Study Design and Participants

**Figure 1:**
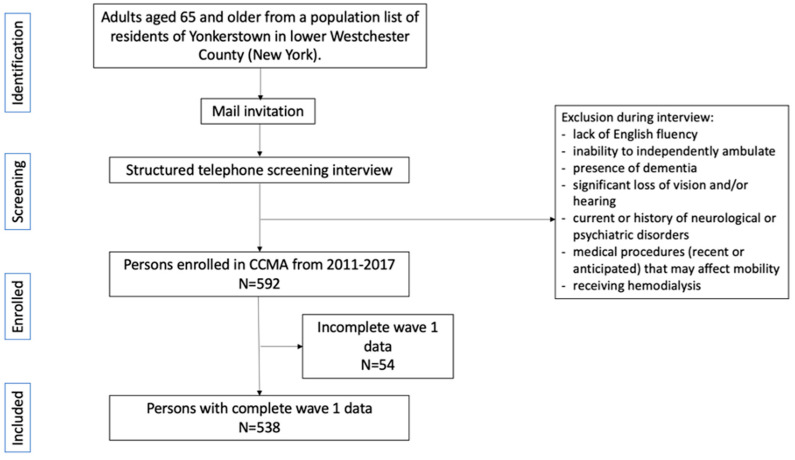
Flow Chart for CCMA Participants Included in Analyses.

We used data collected from 2011 to 2017 of 538 participants enrolled in the Central Control of Mobility in Aging study (CCMA), a prospective cohort study with the primary aim of investigating cognitive predictors of mobility function and disability in the aging population (Figure 1) [28]. Older adults aged ≥ 65 years were identified from a population list of lower Westchester County, New York, invited to participate via mail then telephone, and screened with a structured telephone interview consisting of verbal consent and brief questionnaires assessing medical history, mobility, and validated cognitive screens to exclude dementia. Exclusion criteria consisted of dementia as determined by consensus diagnostic case conference, self-reported cognitive difficulties, inability to walk the length of a room or climb stairs unassisted, and any medical history that would interfere with cognitive or motor testing. Upon enrollment, participants received a structured neurological examination, and comprehensive neuropsychological, cognitive, psychological, and mobility assessments. Clinical and neuropsychological evaluations were repeated annually. Incident diagnoses of dementia and mild cognitive impairment were assigned at consensus case conferences with at least 1 study neurologist and 1 neuropsychologist in attendance, in accordance with criteria of the *Diagnostic and Statistical Manual of Mental Disorders*, fourth edition, using clinical, neuropsychological, and laboratory information. The institutional review board of the Albert Einstein College of Medicine approved experimental procedures, and all participants provided written informed consent.

### 2.2. Apathy

Various apathy scales have been used by clinicians and researchers as there is no one gold standard measure of apathy [29]. Apathy can be measured as a domain of clinically significant depression that is distinct from dysphoria. Participants in the CCMA completed the 30-item Geriatric Depression Scale (GDS) questionnaire, from which the 15-item GDS short form is derived [30]. Since participants did not complete a separate apathy questionnaire, we leveraged the GDS3A, a three-item subscale derived from the GDS15 based on confirmatory factor analysis [31]. The GDS3A (score range 0–3 points) consists of the following three items of the GDS15: (1) Have you dropped many of your activities and interests? Positive response: Yes; (2) Do you prefer to stay at home, rather than going out and doing new things? Positive response: Yes; and (3) Do you feel full of energy? Positive response: No. A score of two or more is positive for the presence of apathy [32].

The GDS3A has a reported sensitivity of 69% and specificity of 85% when compared to the Apathy Evaluation Scale when studied in a cohort of community-dwelling older adults [33]. The GDS3A has been previously utilized by several large cohort studies in examining longitudinal associations between apathy and dementia, as well as in examining cross-sectional neural and inflammatory correlates of apathy [7,19,20,34].

#### 2.2.1. Dysphoric Symptoms of Depression

Dysphoria was defined as endorsing ≥ 2 items on the remaining 12 items of the GDS15. This method of isolating the dysphoric aspect of depression is supported by principal component factors analysis of both the GDS15 and GDS30, where withdrawal/apathy and dysphoria have been identified as distinct domains within the construct of depression [31]. While apathy may occur as a symptom within depression, there is robust evidence that apathy is a distinct entity where diminished affect, social withdrawal, indifference, and diminished goal-directed behavior occur without dysphoria [10,35]. Although the non-apathy domain of depression has typically been described as “depression” or “isolated depression” when used as a covariate in longitudinal studies of apathy and its outcomes, we will be referring to this domain as dysphoria, characterized by emotional distress and negative thought content such as hopelessness, guilt, and pessimism [5,36].

#### 2.2.2. Leisure Activity

At baseline and subsequent study visits, subjects completed questionnaires on their participation in 22 cognitive (reading books or newspapers, writing for pleasure, doing crossword puzzles, etc.) and 24 physical activities (playing tennis or golf, dancing, walking for exercise, etc.). Any physical or cognitive activity with 2 or more participants was additionally categorized as a social activity (n = 11). Participants were asked, “In the past year, how many days per week do you typically engage in this activity?” Frequency of activity participation was scored by activity days per week to compute cognitive (range 0–61), physical (range 0–74), and social (range 0–24) activity scales as previously described [37].

#### 2.2.3. Social Network and Social Behavior

The Social Network Index (SNI) is a commonly used tool to measure social integration. It assesses frequency of interaction within 12 relationship categories and assigns one point for having regular contact, defined as physically seeing or talking on the phone at least once every two weeks, with at least one person per group [38].

We calculated a social behavior composite score by summing the values of the SNI and social activity scale, which was then divided by 12 plus the number of social activities.

We used the Medical Outcomes Study (MOS) social support survey, which assesses perceived availability of social support across the four domains: emotional/informational support, tangible support, affectionate support, and positive social interaction [39].

#### 2.2.4. Covariates

We used the following covariates in our linear regression analyses based on prior reported associations with apathy or dysphoria: age, multimorbidity (2+ comorbidities), dysphoria, gender, and level of education. Previous studies have identified older age, male gender, depressive symptoms, decreased educational years, and multimorbidity as risk factors for apathy in community-dwelling older adults [6,8,14]. Age, multimorbidity, and dysphoria were identified as covariates of apathy from our bivariate analysis of self-reported data at baseline. We measured multimorbidity using a global health score (range 0–10) based on presence or absence of physician-diagnosed depression, diabetes, heart failure, hypertension, angina, myocardial infarction, stroke, PD, chronic obstructive lung disease, and arthritis [40].

#### 2.2.5. Statistical Analysis

We performed bivariate analyses of apathy with demographic factors, the presence of medical and psychiatric comorbidities, and cognitive factors using the independent *t*-test and chi-square test.

We used linear regression models to examine the association between the independent variable apathy, defined by the GDS3A, and the following dependent variables: physical, cognitive, and social leisure activity participation scores, social behavior composite score, Social Network Index, and MOS. We used unadjusted and adjusted linear regression models, using age, multimorbidity, dysphoria, gender, and education as covariates. We reported the unstandardized beta coefficients and the 95% confidence intervals.

All analyses were conducted using SPSS version 29.0 (SPSS Inc., Chicago, IL, USA).

## 3. Results

### 3.1. Participant Characteristics

Of the 538 participants included in the analyses, 55% were female (mean age 76.04 ± 6.49), 80.1% were non-Hispanic white, 66.7% had completed post-secondary education, and 20.4% had 3 or more medical comorbidities. Apathy was present in 29.7% of participants, and 22.1% had dysphoria.

Table 1 presents bivariate analyses of participant characteristics and their associations with apathy. Apathy was associated with older age (t = −2.47, *p* = 0.014), multimorbidity (X2 = 14.08, *p* < 0.001), and dysphoria (X2 = 39.36, *p* < 0.001), but not race/ethnicity, education level, or cognitive performance, including measurements of executive function and short-term memory. Less frequent participation in physical (t = 3.7, *p* < 0.001) and social (t = 3.0, *p* = 0.001), but not cognitive (t = 1.8, *p* = 0.06) activities were associated with apathy. Apathy was also associated with lower SNI (t = 4.0, *p* < 0.001), a lower social behavior composite score (t = 4.1, *p* < 0.001), and a lower MOS overall support score (t = 4.63, *p* < 0.001).

### 3.2. Cross-Sectional Associations with Behavioral Correlates

We explored the association between apathy (defined categorically using the GDS3A subscale) and its behavioral correlates in linear regression models. The presence of apathy was significantly associated with decreased participation in almost two physical activity days per week (−1.688. *p* = 0.003) but not cognitive (−1.094, *p* = 0.252) or social (−0.654, *p* = 0.103) leisure activities (Table 2 and Table 3). Apathy was associated with decreased social behavior based on composite score (−0.055, *p* < 0.001), SNI (−0.478, *p* = 0.003), and MOS (−0.26, *p* = 0.001), after adjusting for age, gender, education level, multimorbidity, and dysphoria (Table 4).

We explored effect modification by gender and dysphoria. After adjusting for confounding factors, the syndrome of apathy had a more meaningful impact on SNI for males (−0.856, *p* < 0.001) compared to females (−0.21, *p* = 0.283) (Supplemental Table S2).

Dysphoria had a similar pattern and was associated with lower participation in physical (−3.277, *p* = 0.002) but not cognitive (−0.135, *p* = 0.897) or social (−0.73, 0.095) activities, decreased social behavior (−0.051, *p* = 0.034), SNI (−0.376, *p* = 0.03), and MOS (−0.28, *p* = 0.001). In stratified analyses by dysphoria status, participants without dysphoria but with apathy had significantly decreased physical leisure activity days (−3.039, *p* = 0.011), MOS scores (−0.342, *p* < 0.001), SNI scores (−0.629, *p* < 0.001), and composite Social Behavior scores (−0.079, *p* = 0.004). Apathy had no significant association with physical leisure, MOS, SNI, and social behavior score for participants with dysphoria (Supplemental Tables S3–S6).

## 4. Discussion

In this study, we explored the cross-sectional associations of apathy with leisure and social engagement in community-dwelling older adults. Our results are consistent with previous reports that apathy is associated with older age, multimorbidity, and dysphoria. We also found that individuals with apathy had diminished social behavior and participated less frequently in physical, but not social or cognitive, leisure activities. When examining specific aspects of social behavior, we found that the presence of apathy was associated with decreased perceived social support and diminished contact within one’s social network, as measured by the MOS and SNI self-report scales, respectively. These associations were independent of dysphoria and remained significant after adjusting for age, gender, education, and multimorbidity. Furthermore, in stratified analyses of participants without dysphoria, these associations remained significant. Additionally, dysphoria exhibited a similar pattern of behavioral correlates that were independent of apathy. After adjusting for the same confounding factors and apathy, the presence of dysphoria was associated with less frequent participation in physical leisure activities only, as well as decreased social behavior and perceived social support. Our findings illustrate that at cross-section, cognitively normal older adults with apathy present with social withdrawal and disinclination for physical activity.

A small number of studies have introduced physical inactivity [26,27] and social dysfunction [41] as behavioral correlates of apathy in community-dwelling older adults. Our study on leisure and social engagement supports these previous findings and adds further clarification that apathy presents with physical and social inactivity in the context of motivation, choice, and reward. Apathy is described as a disorder of impaired motivation with impairments of both initiation and responsiveness in three domains: behavior, cognition, and emotion. Motivated behavior hinges on the choice to act with respect to the costs and benefits associated with the action and is, therefore, influenced by effort sensitivity and reward sensitivity [36,42]. Our findings suggest that for physical leisure, but not cognitive or social leisure activities, the effort of initiating exceeds the reward for those with apathy. The reality that persons with apathy must overcome a greater cost when deciding to initiate physical effort has been previously suggested in functional brain imaging studies that show increased frontal lobe activation during decision making [43]. Our results also suggest that apathy presents with defects in both initiation and responsiveness regarding social engagement. The association of apathy with decreased SNI, which measures how many different types of social contacts an individual communicates with on a regular basis, suggests diminished initiative in terms of behavior. The observed decrease in MOS, which assesses the perceived availability of social support, suggests diminished emotional response to social relationships.

Our findings are cross-sectional but may indicate potential clinical implications for targeted interventions in community-dwelling older adults with apathy. We show that apathy in cognitively normal older adults presents with decreased social engagement and physical leisure activity, two protective factors against dementia. The existing literature suggests that social engagement, community support, and regular physical activity decreases the risk of cognitive decline and incident dementia in healthy older adults [44,45,46,47]. Previous studies have also shown that late-life social isolation is also associated with physical inactivity and a more rapid decline in motor function [48,49], though the mechanism for this association remains unclear. Social isolation and loneliness have been shown to correlate with increased markers of chronic inflammation [48,50], while physical activity has been shown to reduce inflammatory markers in older adults, and this reduction is often associated with better cognitive performance [47]. Both social and physical activity depend on the integration of sensory, motor, and cognitive neural functions involved in the planning and execution of goal-directed behavior [49,51]. Additionally, decreased connectivity between the neural circuits involved in motivated behavior have been observed in neuroimaging studies of apathy [52,53]. The extant literature and our findings taken together suggest that social isolation, physical activity, and apathy may all be related, and that apathy itself is not just a risk factor of dementia but may perpetuate cognitive decline through decreased physical activity and social engagement. However, we would require more longitudinal studies to better understand the causal relationship.

There are some limitations of our study. We analyzed data collected from an existing cohort study and, therefore, relied on the GDS3A subscale to measure apathy from existing GDS15 data. Although the GDS3A subscale measurement of apathy has less sensitivity and specificity compared to scales developed to specifically measure apathy [33], this subscale is well-established and has previously been used in large cohort studies of apathy [20,33,34]. The use of self-report scales is susceptible to social desirability and recall biases. However, this would be less likely given that participants were asked to recall their activity engagement during the past month. Furthermore, associations that have been identified with apathy and dysphoria in our study population, the CCMA participants, may not be generalizable to ambulatory geriatric populations that are more racially, ethnically, and educationally diverse. This is a larger issue reflecting the limitations of traditional cohort studies, which often rely on a battery of testing over several days and may be prohibitive to those who suffer from higher levels of apathy. Finally, our study utilizes cross-sectional data analysis, which does not describe the directionality of the relationships between apathy and its behavioral correlates. Future investigations should clarify the direction of the relationship between apathy and social isolation, and apathy and physical activity. The adverse health effects of late-life social isolation and loneliness, including the worsening of depression and anxiety [54] and increased mortality [55], are especially salient given the unprecedented social isolation resulting from the COVID-19 pandemic [56]. Therefore, it is of interest to clarify whether social isolation causes apathy, and if apathy decreases engagement in physical activity and social engagement. It would be of further interest to delineate which specific physical leisure activities are correlated with the presence of apathy as these may also be targets for interventions.

Future analyses could include subgroup analyses of these associations by cognitive status, functional status, and by race ethnicity to explore effect modification. Future study designs should also be more inclusive and accessible to people with higher levels of apathy and functional impairment, and less reliant on self-report and formally tested data. Such methods include utilizing home visits, collateral informants, studying cohorts from more diverse patient populations, and directly observing behavior using actigraphy.

## 5. Conclusions

Our study contributes to the understanding of the presentation and clinical significance of apathy and focuses on aspects of apathy that have been minimally explored. We explored associations between apathy and volitional engagement in rewarding behavior in cognitively normal community-dwelling older adults using validated scales that have been previously applied in our cohort. Few studies have examined the behavioral correlates of apathy, or the presentation of apathy in cognitively normal community-dwelling older adults. Even fewer studies have studied the associations between apathy and leisure. Our findings help contextualize the role of motivation and reward in apathy, as it relates to previous findings regarding apathy, physical activity, and social engagement. This study demonstrates that apathy, independent of dysphoria, presents with reduced physical leisure participation and social engagement, which may provide a simplified way for clinicians and caregivers to identify apathy in the future.

## Figures and Tables

**Table 1:** Baseline Characteristics by Apathy.

Variable	No Apathy (N = 378) Mean (±SD)	Apathy (N = 160) Mean (±SD)	Statistic	*p*-Value
Age, years	76 (6.33)	77 (6.74)	t = −2.47	0.014 *
Female Sex, N (%)	207 (55)	90 (56)	X2 = 0.101	0.751
Race/Ethnicity, N (%)			X2 = 3.45	0.327
Non-Hispanic White	310 (82)	121 (75)		
Non-Hispanic Black	54 (14)	33 (21)		
Hispanic	8 (2)	3 (2)		
Education, N (%)			X2 = 3.04	0.219
HS grad	109 (29)	39 (24)		
Greater than HS	251 (66)	108 (68)		
Chronic medical comorbidities, N (%)			X2 = 14.08	<0.001 *
no med dx	69 (18)	16 (10)		
1–2 med dx	246 (65)	97 (61)		
3–4 med dx	63 (17)	47 (29)		
Dysphoria, N (%) ^a^	56 (15)	63 (40)	X2 = 39.36	<0.001 *
Cognition ^b^				
Trails delta (0–310)	80.8 (55.9)	79.8 (53.1)	t = 0.187	0.852
RBANS				
Delayed Recall (65–126)	93.1 (10.9)	92.7 (9.4)	t = 0.478	0.633
Global Cognition Score (49–173)	118.4 (17.2)	118.3 (15.4)	t = −0.151	0.88
Leisure Activity				
Cognitive (0–61)	27.2 (10.2)	25.3 (10.0)	t = 1.8	0.06
Physical (0–74)	21.4 (9.9)	17.7 (10.4)	t = 3.7	<0.001 *
Social (0–24)	6 (4.2)	4.8 (4.1)	t = 3.0	0.001 *
Social Factors				
Social Network Index (0–12)	5.4 (1.5)	4.7 (1.8)	t = 4.0	<0.001 *
Composite Social Behavior Score (0–1.57)	0.054 (0.23)	0.045 (0.23)	t = 4.1	<0.001 *
MOS Social Support Survey-Overall Support (0–5)	4.17 (0.79)	3.79 (0.9)	t = 4.63	<0.001 *

Note: * *p* < 0.05 was considered significant; Apathy defined as 2+ positive items on GDS3A subscale; ^a^ Dysphoria defined as endorsing ≥ 2 items in the remaining 12 Items of the GDS; ^b^ corrected for age and education.

**Table 2:** Linear Regression of the Association of Apathy with Cognitive and Physical Leisure Activities.

Independent Variable	Cognitive Leisure		Physical Leisure	
	Unstd Beta (95% CI)	*p*-Value	Unstd Beta (95% CI)	*p*-Value
Model 1	−1.802 (−3.68, 0.079)	0.06	−2.786 (−5.58, −1.87)	<0.001 *
Model 2	−1.728 (−3.622, 0.02)	0.074	−2.377 (−5.31, −1.60)	<0.001 *
Model 3	−1.206 (−2.99, 0.58)	0.184	−2.42 (−5.34, −1.53)	<0.001 *
Model 4	−1.125 (−2.94, 0.69)	0.224	−2.445 (−5.30, −1.52)	<0.001 *
Model 5	−1.094 (−2.97, 0.78)	0.252	−1.688 (−4.60, −0.73)	0.003 *

Note. Model 1: Apathy; Model 2: Model 1 and adjusted for age; Model 3: Model 2 and adjusted for gender and education; Model 4: Model 3 and adjusted for number of comorbidities; Model 5: Model 4 and adjusted for dysphoria; * *p* < 0.05.

**Table 3:** Linear Regression of the Association of Apathy with Social Leisure and Social Behavior.

Independent Variable	Social Behavior Score		Social Leisure	
	Unstd Beta (95% CI)	*p*-Value	Unstd Beta (95% CI)	*p*-Value
Model 1	−0.094 (−0.12, −0.07)	<0.001 *	−1.214 (−1.81, −0.89)	0.002 *
2	−0.082 (−0.11, −0.01)	<0.001 *	−1.014 (−1.44, −0.53)	0.009 *
3	−0.073 (−0.01, −0.05)	<0.001 *	−0.871 (−1.44, −0.53)	0.023 *
4	−0.071 (−0.01, −0.05)	<0.001 *	−0.819 (−1.40, −0.49)	0.035 *
5	−0.055 (−0.08, −0.03)	<0.001 *	−0.654 (−1.19, −0.25)	0.103

Note. Model 1: Apathy; Model 2: Model 1 and adjusted for age; Model 3: Model 2 and adjusted for gender and education; Model 4: Model 3 and adjusted for number of comorbidities; Model 5: Model 4 and adjusted for dysphoria; * *p* < 0.05.

**Table 4:** Linear Regression of the Association of Apathy with Social Network Index and MOS Social Support.

Independent Variable	Social Network Index		MOS Social Support	
	Unstd Beta (95% CI)	*p*-Value	Unstd Beta (95% CI)	*p*-Value
Model 1	−0.644 (−0.94, −0.35)	<0.001 *	−0.38 (−0.53, −0.23)	<0.001 *
2	−0.62 (−0.91, −0.32)	<0.001 *	−0.35 (−0.5, −0.19)	<0.001 *
3	−0.603 (−0.90, −0.31)	<0.001 *	−0.35 (−0.5, −0.19)	<0.001 *
4	−0.564 (−0.87, −0.26)	<0.001 *	−0.32 (−0.48, −0.17)	<0.001 *
5	−0.478 (−0.79, −0.17)	0.003 *	−0.26 (−0.42, −0.1)	0.001 *

Note. Model 1: Apathy; Model 2: Model 1 and adjusted for age; Model 3: Model 2 and adjusted for gender and education; Model 4: Model 3 and adjusted for number of comorbidities; Model 5: Model 4 and adjusted for dysphoria; * *p* < 0.05.

## Data Availability

In line with data sharing guidelines set by the NIH, all data, analytic methods, and study materials will be shared to other investigators upon request to the corresponding author. This study was not preregistered.

## Supplementary Materials

The following supporting information can be downloaded at: https://www.mdpi.com/article/10.3390/ijerph22071138/s1. Supplemental Table S1: Linear Regression of the Association of Apathy with MOS, by Gender, Supplemental Table S2: Linear Regression of the Association of Apathy with SNI, by Gender, Supplemental Table S3: Linear Regression of the Association of Apathy with Physical Leisure, by Dysphoria, Supplemental Table S4: Linear Regression of the Association of Apathy with MOS, by Dysphoria, Supplemental Table S5: Linear Regression of the Association of Apathy with SNI, by Dysphoria, Supplemental Table S6: Linear Regression of the Association of Apathy with Social Behavior Score, by Dysphoria.
