# Supplementary material for: Diminished Social and Leisure Engagement in Community Dwelling-Older Adults with Apathy
Source: Int J Environ Res Public Health. 2025 Jul 18;22(7):1138. doi: 10.3390/ijerph22071138 (PMC12294408; doi:10.3390/ijerph22071138)
Supplement: Supplementary file 1 [file ijerph-22-01138-s001.zip › ijerph-3703824-supplementary.pdf]

Supplemental Table 1: Linear Regression of the Association of Apathy with MOS, by Gender				
**Independent Variable**	Male		**Female**	
	Unstd Beta (95% CI)	P-value	Unstd Beta (95% CI)	**P-value**
**Model 1**	-0.445 (-0.674 , -0.216)	**<.001***	-0.326 (-0.531 , -0.12)	**0.002***
**Model 2**	-0.431 (-0.655 , -0.207)	**<.001***	-0.284 (-0.491 , -0.078)	**0.007***
**Model 3**	-0.424 (-0.65 , -0.199)	**<.001***	-0.288 (-0.495 , -0.081)	**0.007***
**Model 4**	-0.416 (-0.646 , -0.186)	**<.001***	-0.255 (-0.465 , -0.045)	**0.017***
**Model 5**	-0.351 (-0.585 , -0.117)	**0.003***	-0.187 (-0.403 , 0.028)	**0.089**

***Note*. Model 1: Apathy; Model 2: Model 1 and adjusted for age; Model 3: Model 2 and adjusted for gender and education; Model 4: Model 3 and adjusted for number of comorbidities; Model 5: Model 4 and adjusted for dysphoria**
****p*<0.05**

**Supplemental Table 2:** *Linear Regression of the Association of Apathy with SNI, by Gender*				
	Male		Female	
Independent Variable	Unstd Beta (95% CI)	P-value	Unstd Beta (95% CI)	P-value
Model 1	-0.827 (-1.305 , -0.349)	**<.001***	-0.499 (-0.872 , -0.125)	**0.009***
Model 2	-0.813 (-1.29 , -0.335)	**<.001***	-0.478 (-0.858 , -0.099)	**0.014***
Model 3	-0.829 (-1.309 , -0.348)	**<.001***	-0.453 (-0.828 , -0.079)	**0.018***
Model 4	-0.909 (-1.397 , -0.422)	**<.001***	-0.33 (-0.703 , 0.044)	0.083
Model 5	-0.856 (-1.356 , -0.355)	**<.001***	-0.21 (-0.594 , 0.174)	0.283

Note. Model 1: Apathy; Model 2: Model 1 and adjusted for age; Model 3: Model 2 and adjusted for gender and education; Model 4: Model 3 and adjusted for number of comorbidities; Model 5: Model 4 and adjusted for dysphoria
**p*<0.05

Supplemental Table 3: Linear Regression of the Association of Apathy with Physical Leisure, by Dysphoria				
	No Dysphoria		Dysphoria	
Independent Variable	Unstd Beta (95% CI)	P-value*	Unstd Beta (95% CI)	P-value*
Model 1	-3.358 (-5.654 , -1.063)	**0.004***	-1.994 (-5.442 , 1.454)	0.254
Model 2	-3.059 (-5.379 , -0.74)	**0.01***	-2.199 (-5.58 , 1.182)	0.2
Model 3	-3.087 (-5.408 , -0.767)	**0.009***	-2.164 (-5.532 , 1.204)	0.206
Model 4	-3.039 (-5.374 , -0.703)	**0.011***	-2.33 (-5.86 , 1.2)	0.194

Note. Model 1: Apathy; Model 2: Model 1 and adjusted for age; Model 3: Model 2 and adjusted for gender and education; Model 4: Model 3 and adjusted for number of comorbidities
**p*<0.05

Supplemental Table 4: *Linear Regression of the Association of Apathy with MOS, by Dysphoria*				
	No Dysphoria		Dysphoria	
Independent Variable	Unstd Beta (95% CI)	P-value*	Unstd Beta (95% CI)	P-value*
Model 1	-0.391 (-0.572 , -0.211)	**<.001***	-0.079 (-0.403 , 0.245)	0.629
Model 2	-0.335 (-0.515 , -0.156)	**<.001***	-0.09 (-0.414 , 0.234)	0.583
Model 3	-0.344 (-0.524 , -0.164)	**<.001***	-0.089 (-0.414 , 0.237)	0.591
Model 4	-0.342 (-0.523 , -0.16)	**<.001***	0 (-0.335 , 0.335)	0.999
Note. Model 1: Apathy; Model 2: Model 1 and adjusted for age; Model 3: Model 2 and adjusted for gender and education; Model 4: Model 3 and adjusted for number of comorbidities<br>**p*<0.05				

**Supplemental Table 5:** ***Linear Regression of the Association of Apathy with SNI, by Dysphoria***				
	No Dysphoria		Dysphoria	
Independent Variable	Unstd Beta (95% CI)	P-value	Unstd Beta (95% CI)	P-value
Model 1	-0.694 (-1.058 , -0.329)	**<.001***	-0.173 (-0.739 , 0.394)	0.547
Model 2	-0.645 (-1.014 , -0.277)	**<.001***	-0.169 (-0.739 , 0.4)	0.557
Model 3	-0.639 (-1.009 , -0.268)	**<.001***	-0.154 (-0.715 , 0.406)	0.586
Model 4	-0.629 (-1.002 , -0.256)	**<.001***	0.001 (-0.578 , 0.579)	0.998
Note. Model 1: Apathy; Model 2: Model 1 and adjusted for age; Model 3: Model 2 and adjusted for gender and education; Model 4: Model 3 and adjusted for number of comorbidities<br>**p*<0.05				

Supplemental Table 6: Linear Regression of the Association of Apathy with Social Behavior Score, by Dysphoria				
	No Dysphoria		Dysphoria	
Independent Variable	Unstd Beta (95% CI)	P-value	Unstd Beta (95% CI)	P-value
Model 1	-0.104 (-0.157 , -0.051)	**<.001***	0.002 (-0.071 , 0.075)	0.959
Model 2	-0.086 (-0.139 , -0.033)	**0.001***	0 (-0.073 , 0.072)	0.991
Model 3	-0.08 (-0.133 , -0.028)	**0.003***	0.004 (-0.061 , 0.069)	0.907
Model 4	-0.079 (-0.131 , -0.026)	**0.004***	0.02 (-0.047 , 0.087)	0.559
Note. Model 1: Apathy; Model 2: Model 1 and adjusted for age; Model 3: Model 2 and adjusted for gender and education; Model 4: Model 3 and adjusted for number of comorbidities; Model 5: Model 4 and adjusted for dysphoria<br>**p*<0.05				
